# Recent Developments in Shape Memory Elastomers for Biotechnology Applications

**DOI:** 10.3390/polym14163276

**Published:** 2022-08-11

**Authors:** Supitta Suethao, Thridsawan Prasopdee, Kwanchai Buaksuntear, Darshil U. Shah, Wirasak Smitthipong

**Affiliations:** 1 Specialized Center of Rubber and Polymer Materials in Agriculture and Industry (RPM), Department of Materials Science, Faculty of Science, Kasetsart University, Bangkok 10900, Thailand; 2 Department of Mechanical Engineering, Faculty of Engineering, King Mongkut’s University of Technology Thonburi, Bangkok 10150, Thailand; 3 Centre for Natural Material Innovation, Department of Architecture, University of Cambridge, Cambridge CB2 1PX, UK; 4 Office of Research Integration on Target-Based Natural Rubber, National Research Council of Thailand (NRCT), Bangkok 10900, Thailand

**Keywords:** shape memory, smart polymers, self-healing, elastomers, biotechnology

## Abstract

Shape memory elastomers have revolutionised the world since their introduction in the 20th century. The ability to tailor chemical structures to produce a family of materials in wide-ranging forms with versatile properties has propelled them to be ubiquitous. Recent challenges in the end-of-life management of polymeric materials should prompt us to ask, ‘what innovations in polymeric materials can make a strong case for their use as efficient materials?’ The development of smart elastomers that can acquire, convey, or process a stimulus (such as temperature, pressure, electromagnetic field, moisture, and chemical signals) and reply by creating a useful effect, specifically a reversible change in shape, is one such innovation. Here, we present a brief overview of shape memory elastomers (SMEs) and thereafter a review of recent advances in their development. We discuss the complex processing of structure-property relations and how they differ for a range of stimuli-responsive SMEs, self-healing SMEs, thermoplastic SMEs, and antibacterial and antifouling SMEs. Following innovations in SEMs, the SMEs are forecast to have significant potential in biotechnology based on their tailorable physical properties that are suited to a range of different external stimuli.

## 1. Introduction

Smart materials, also known as intelligent or responsive materials, are a useful name for a broad group of different chemical substances. A common characteristic is that one or more properties may change significantly, even reversibly, in response to the specific stimulus input of preset form and extent over short or appropriate timescales and return to its original shape as the stimulus is taken away. The stimulus could be strain, stress, temperature, pressure, electric and magnetic fields, irradiation, or chemicals. Examples of smart materials include coatings that transform colour owing to the presence of chemicals, materials that have a shape memory at specific temperatures and paint that self-heals when it is scratched [1,2,3,4,5,6,7]. Smart materials can acquire, convey, or process a stimulus and reply by creating a ‘useful’ effect. This ability offers opportunities for responsive materials in applications such as actuation, shape memory, and sensing in industries including biomedicine, aerospace, textiles, and so on [8,9,10,11,12,13,14].

Shape memory polymers (SMPs) can memorise an original shape and can be tailor-made to some temporary shapes, whereas they naturally return to their original permanent shape from the temporary deformations upon simultaneous disclosure to various (even multiple) stimuli without additional mechanical effort. Typically, SMPs exhibit at least two phases: the first is a stable form that stabilises the SMP and is responsible for the retention of the original shape and the second that responds to the external trigger [15,16,17,18,19,20,21,22,23,24,25]. SMPs can be categorized into one of four types depending on their chemical structure: thermosets, rubbers, thermoplastics, and block copolymers. This classification decides the mechanisms of the shape memory of the SMP [26,27,28,29,30].

**Figure 1:**
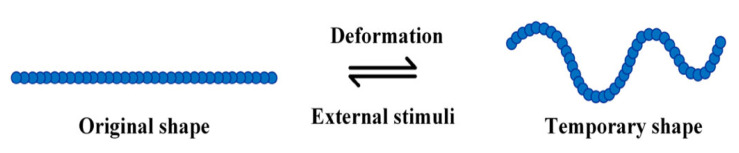
Characteristic behaviour of shape memory elastomers: (i) deformation of the material transforms the original ‘equilibrium’ shape into a temporary shape, and (ii) external stimuli trigger and affect the return to the original shape from the temporary shape.

Generally, SMPs are rigid and non-elastomeric at the application temperature [31,32,33,34]. In contrast, shape memory elastomers (SMEs) are softer and more elastomeric than SMPs since elastomers are polymers with the property of “elasticity”, typically having low tensile strength and high elongation at breakage. Therefore, elastomers can withstand high elastic deformation without rupture. Moreover, elastomers are relatively soft and deformable at ambient temperatures due to their low glass transition temperatures (*T*_g_) [35,36,37,38,39]. Some key differences between polymers and elastomers are summarised in Table 1. The high viscoelasticity of elastomers leads to a strong influence of ‘transformation temperature’ on the shape memory properties of an SME. The transformation temperature is typically 15–20 °C higher than the *T*_g_, though it may be in the vicinity of or even below that of the *T*_g_. When passing the *T*_g_ from the glassy to the rubbery state, it is known that the network deformability goes through a maximum [24]. A sample model of an SME is presented in Figure 1.

**Figure 2:**
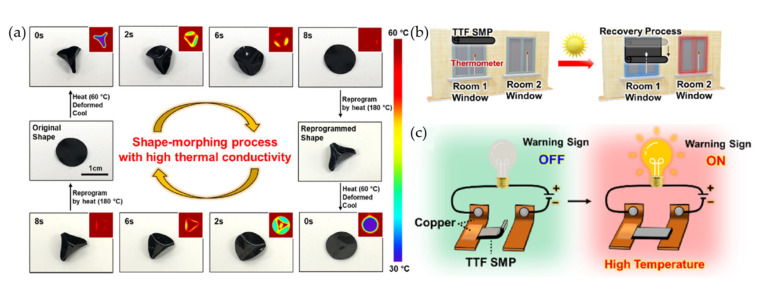
The shape memory process of tetrathiafulvalene elastomer networks (TTF SME) and their applications; (**a**) photograph and thermographic images of TTF SME, (**b**) the sunshade thermo-responsive, and (**c**) high-temperature warning sensor [43]. Adapted with permission from [43]. Copyright 2022 American Chemical Society.

SMEs are elastic polymer networks, which are an appearing type of active elastomer, and have a dual-shape (or multi-shape) architecture. Generally, SMEs exhibit two types of structure: original shape and deformed (temporary) shape, which can be switched reversibly under specific conditions. A category of smart materials, SMEs react to external stimuli such as temperature, pressure, electromagnetic fields, chemicals (and pH), water, light, and so on. The responsive physical properties could be stiffness, shape, and damping, among others [1,9,14,24,40,41,42]. A recent SME was developed by Rim et al. (2022) [43]. Figure 2a shows the shape memory process of tetrathiafulvalene elastomer networks (TTF SME) by heat. The results revealed that the shape recovery of TTF SME was reached within 8 s due to its good thermal conductivity. Furthermore, the TTF SMEs could be applied in smart materials such as thermoactuating smart sunshades and warning sensors, as shown in Figure 2b,c [43]. Table 2 lists examples of SMEs, their chemical nature and potential triggers, and their applications studied in the literature. We explore these in more detail in this review.

SMEs such as polyurethane and natural rubber typically comprise two regions: amorphous and crystalline. The amorphous region supports the stabilisation and retention of the original shape, and then the deformation of this region induces shape recovery. In contrast, the crystalline region is responsible for obstructing shape recovery until a critical condition is met [23,41,56,57].

Current applications of SMEs are principally in the consumer products sector (Table 3). In the present era of health and fitness, comfort fitting is becoming an increasingly important consideration in choosing personal products such as clothing, footwear, insoles, and orthotic devices [58]. For footwear, the demand for more comfort and functionality makes the characteristics of SMEs attractive and appealing to footwear design [59]. In 2021, the International Trade Centre revealed that the sale of sports shoes had grown 31% compared to 2020, with the market worth USD 2320 million, and bedding sales had grown 17% compared to 2020, with the market worth USD 115 million. Moreover, for thermoplastic elastomers, a market research report by MarketsandMarkets™ revealed that the market was worth USD 5.1 billion in 2021, with a compound annual growth rate of 5.8% between 2021 and 2026 (Table 3).

This mini review discusses recent advances in the development of SMEs in a large range of applications, particularly biomedical and environmental applications, based on their tailorable physical properties that are suited to a range of different external stimuli.

## 2. Thermodynamic Aspects Governing SMEs

**Figure 3:**
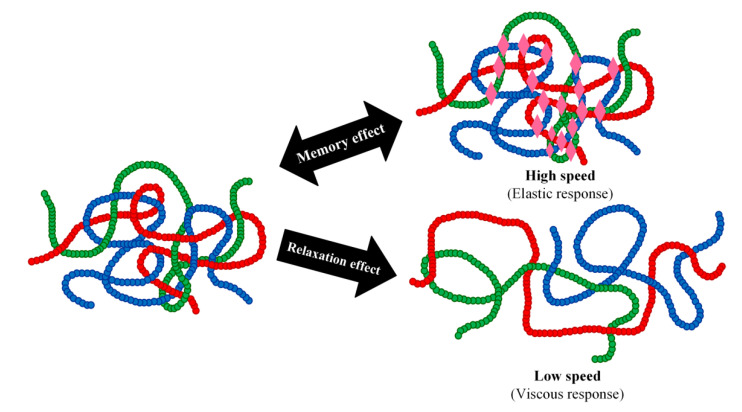
Entangled elastomer chains (with different colours illustrating different chains) respond to externally applied force in different ways. If we quickly stretch an elastomer, the entangled chains will make provisional knots that oppose the movement of the chains. The elastomer displays an elastic response (memory effect). On the other hand, if we slowly stretch the entangled chains, they have time to relax and restrain and rearrange themselves. The elastomer chains flow similar to a liquid, thereby showing a viscous response. Thus, the response of an elastomer to external stress is always the result of the balance between the rate of molecular agitation (which increases with high temperatures) and the rate of external stress (which increases with high frequencies or short times). These behaviours are the origin of one of the characteristic properties of polymers: the law of time–temperature superposition.

From an elastomer transition state point of view, all movements of the elastomer molecules are fixed in the glassy region of the elastomer’s character (below its glass transition temperature). When the temperature is increased, the rotation surrounding the elastomer bonds also increases approaching the rubbery region, indicating that the elastomer molecules are close to entangled. Most of the macromolecules form a highly coiled conformation with the maximum entropy based on the Boltzmann equation [63]. In the rubbery state, an elastomer with sufficient molecular weight can be stretched along the alignment of the external force. When the force is applied at a high speed or over short periods of time, the local entanglement of the elastomer molecules can block the movement of the molecule. Consequently, the elastomer will recover to its original shape once the applied force is taken away, exhibiting what is known as a viscoelastic response. In this way, the elastomer with a randomly coiled state possesses a type of memory of its initial state thereby exhibiting a memory effect. In contrast, when the applied force is applied at a low speed or over long periods of time, a relaxation process will take place. This relaxation is due to the slipping and disentangling of the elastomer chains from each other into new positions, which enables the molecules to form other random coil conformations with favourable entropy (Figure 3). The described slipping or flow of the elastomer molecules under force can be prevented by crosslinking the molecules. Chemically crosslinked elastomers form insoluble elastomers; their structure is linked during the crosslinking process and then cannot be transformed afterwards [64].

Therefore, molecular mobility is the main cause of the shape memory effect for polymers as mentioned before. For example, Oikonomou et al. (2021) [65] studied the relationship between free volume and molecular mobility using the Couchman−Karasz and Gordon−Taylor equations, which are numerical approaches to describing molecular mobility in poly(vinyl alcohol) and poly(vinyl pyrrolidone) blends. The results revealed that molecular mobility increased with the increasing free volume in the blend system.

During the transition of a shape memory elastomer from its original shape to a temporary shape and then its return to the original shape based on the stimuli-response environment, there are two types of phases or domains in the system [66]. This is related to the phase diagrams, which are a graphical representation of the various material domains of stability at equilibrium. Phase diagrams are most commonly constructed in temperature–pressure–composition space, or Gibbs free energy composition space. Other coordinate systems, though not yet as widely used, may find increasing practical applications, especially in predicting changes in the internal structure of shape memory. A system that is equilibrated in some initial domain on the map and then placed in another domain with a different equilibrium structure, such as by simply changing its temperature, undergoes a series of microstructure transformations that take it towards its new equilibrium state. Microstructures with optimum properties can be selected by interrupting this process and quenching (i.e., rapid cooling) in the structure, which is then the state in which the shape memory elastomer is used.

A system is considered to be unary (single phase) if for the range of states under study it consists of a single chemical component. Each of the elements forms a unary system over its full range of existence. Molecular compounds such as synthetic polyisoprene (*cis*–1,4–polyisoprene) may be treated as unary systems over most of the range of temperatures and pressures normally encountered in the laboratory. Under conditions in which it may decompose or change to form significant quantities of other molecules, it cannot be treated as a unary system. From a thermodynamic point of view, a system is homogeneous if it consists of a single phase. More specifically, when a system consists of a single phase, its intensive properties are similar. A heterogeneous system consists of more than one phase. Some of its intensive properties exhibit discontinuities at the boundaries between the phases in a heterogeneous system. In the case of SMEs, the thermodynamic equilibrium is not always reached with regard to compatibility (as opposed to miscibility). Miscibility always relates to small molecules. The definition of compatibility is linked to the properties of the mixture as natural latex (*cis*–1,4–polyisoprene molecules with non–rubber components); if the response of a mixture to the input force is one model or one characteristic, the mixture is compatible. Therefore, thermodynamics is a significant property-governing character in the investigation and development of shape memory elastomers [67].

## 3. Stimuli-Responsive Shape Memory Elastomers

SMEs transform their shape in a designed way under appropriate external stimuli; there are several ways that this novel functionality could potentially play an important role [68,69,70,71]. An example of an SME is an elastomer reinforced by a semi-crystalline fibre network; the elastomeric matrix gives background elasticity, whereas the reinforced fibre can change between the solid phases and melt phases as a function of temperature. Therefore, it offers a reversible phase that possesses a shape memory effect. Shape fixing, or fixity, refers to the ability to retain a temporary state or the ability to fix the temporary deformation and thus store strain energy, whereas shape recovery describes the ability to recover the original shape. SME composites offer a new prototype for the advancement of a large order of elastomeric composites that exhibit the crystal–melt transformation to reach the shape memory phenomenon [72,73,74].

SMEs can be thermo-responsive, which means that any temporary transition can be removed, and their original shape can be returned under a specific condition or temperature. These elastomers always create network structures (either chemical crosslinking or physical crosslinking) that define their original shapes. Their shape memory effect is often related either to melting or glass transition temperature [75,76,77].

Biodegradable SMEs have high performance in the biomedical field. A thermally induced polyurethane SME based on poly(*ε*–caprolactone) (PCL) and poly(L–lactic acid) (PLLA) was studied by Chien and co-workers and the PCL- and PLLA-blended ratio in polyurethane was adjusted for recovery behaviour [78]. The temperature of polymerization affected the crystallinity and miscibility of PCL and PLLA molecules with different melting points. They found that the polyurethane chains are flexible at high temperatures.

**Figure 4:**
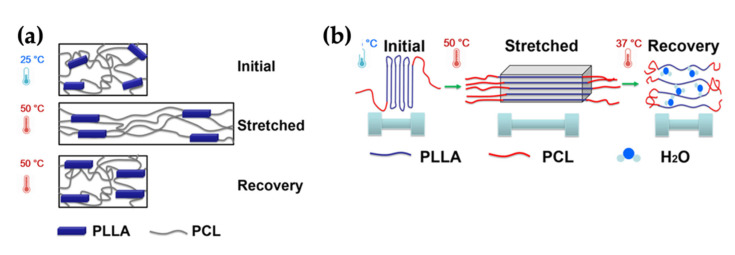
(**a**) Crystalline PLLA presented a random distribution and amorphous PCL exhibited a soft character before extending. After deformation, the crystal PLLA was stretched by arranging it to the stretched alignment and soft PCL was also arranged. When the sample was heated at 50 °C, the amorphous PCL returned to its original shape. (**b**) Polyurethane SMEs were developed to apply as a biomaterial with shape memory behaviour at 37 °C under water. The crystal PLLA was aligned to create the crystalline structure, and the hydrogen bonding and van der Waal force could play a major role in forming the stretched shape. After the sample was cooled down under water, the crystalline elastomer chains could be softened by the water molecules. Adapted with permission from [78]. Copyright 2017 American Chemical Society.

Figure 4 shows the mechanism of the recovery process at 50 °C in air and 37 °C under water. For the synthesised polyurethanes, the strain-induced crystallisation of PLLA was studied as the temporary shape and the amorphous PCL oligodiol was considered for the recovered shape. The shape recovery ratio at 37 °C under water was almost 100% and the recovery was quick. Thus, this type of SME exhibits a thermally induced shape memory behaviour and could offer good applications in biodegradable SMEs.

Du and co-workers [79] investigated the new polyolefin elastomer (POE)/lauric acid (LA)/carbon black (CB) nanoparticle composite with shape memory behaviour. The composite system was developed by melt blending; POE possessed great elasticity and LA was utilised to facilitate the shape changing. CB nanoparticles were used as reinforcing fillers because of their high chemical stability and improvement in the recovery force. This SME can be modified into a temporary shape with good shape recovery and quickly responds to thermo- and electro-stimuli.

Since the crystalline LA fixed the temporary shape, the crystal LA can be dissolved by a suitable solvent to trigger shape recovery. When the temporary shape was immersed into ethanol at room temperature, it reached close to complete recovery in almost 30 min. Thus, there was no need to heat the sample for shape recovery. Moreover, the SME can control its shapes with solvent stimuli and transform chemical responses into mechanical responses [79]. Therefore, SMEs triggered using diffused water give an advantage against the heating sample to avoid heat degradation [51].

**Figure 5:**
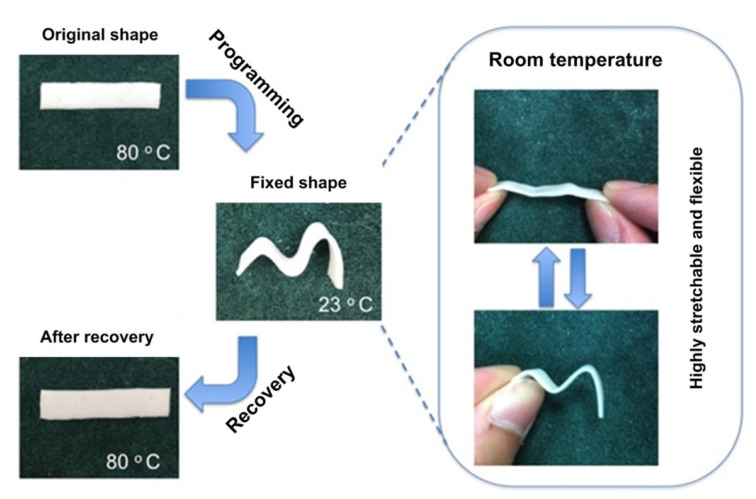
Example behaviour of an SME showed a flat sample in its original state at 80 °C; the sample transformed into a deformed shape at room temperature. After that, heating again to 80 °C resulted in a full return to the original flat shape from the deformed shape. Reprinted from [40], Copyright 2017, with permission from Elsevier.

Another example is the preparation and characterisation of a water-triggered shape memory elastomer poly(ε–caprolactone) (PCL)—poly(ethylene glycol) (PEG)-based thermoplastic polyurethanes (TPUs). When the sample was immersed in water, the hydrophilic PEG swelled, indicating great elongation at a break of almost 700%. With the dry samples, just the aligned PEG returned to the original shape resulting in complete shape recovery. The speed of recovery of this material was investigated by varying the thickness of elastomeric films [50]. Interestingly, the typical behaviour of SME are presented in Figure 5, which exhibited full recovery from the zig-zag shape to the original shape after heating.

A diketopyrrolopyrrole-based conjugated polymer (PDPP3T) was developed with polycaprolactone–co–poly(urethane/urea) (PCL–PU) to reach the light-induced reconfigurable (shape memory behaviour) observed by Zhang et al. [53]. The PDPP3T showed a strong near-infrared absorption with few emissions in the solid state, and the PCL–PU elastomer represented outstanding light-induced shape recovery close to 100% via photothermal effects under 808 nm laser irradiation.

An ideal stimulus must be considered for biomedical applications based on the physiological environment. Various physiological pH are present in the body at different locations [44]. An SME of polyurethane with pH-sensitive shape memory behaviour was developed by Song et al. [80]. Polyethylene glycol (PEG), dimethylol propionic acid (DMPA), and 4,4′–diphenylmethane diisocyanate (MDI) were used to synthesise the PEG–i–MDI–DMPA, (i = 20%, 30%, 40%), where i presents the PEG content in the elastomer. The results showed that the carboxylic dimer in PEG–30%–MDI–DMPA influenced the pH values to encourage the pH-sensitive SME. On the contrary, PEG–20%–MDI–DMPA and PEG–40%–MDI–DMPA represented a low shape memory effect because they exhibited either higher or lower carboxylic contents.

Moreover, Wang et al. [81] used nano-Fe_3_O_4_ particles in the poly(styrene–*b*–butadiene–*b*–styrene) copolymer (SBS)/liner low-density polyethylene (LLDPE) blends that can be developed by the magnetically sensitive SME. The results revealed that the dispersed Fe_3_O_4_ nanoparticles endowed the homogeneous heat generation transfer in the rotating magnetic field and gave the SME a great magnetically responsive shape memory behaviour. This SME also represented great thermal–mechanical performance.

## 4. Self-Healing Shape Memory Elastomers

Self-healing elastomers can be developed to become smart materials because they offer structural restoration and shape recovery after damage, which can improve the product’s lifespan and reduce maintenance costs [82,83]. Self-healing is the ability of a biological or artificial system to fully or partially spontaneously repair any damage arising when certain trigger conditions are met [84]. However, this ability is rare in typically artificial materials because their molecular chains cannot move over the damages to reform bonds [85].

**Figure 6:**
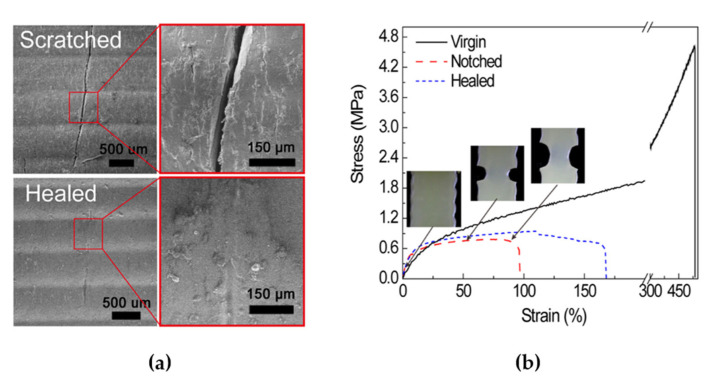
SEM micrographs of self-healing behaviour of printed semi-interpenetrating polymer network elastomer composites show scratched and healed surfaces (**a**). The healing strain was relatively low compared with the virgin sample (**b**). The fracture energy can be calculated by the area under the stress–strain curve. After healing, the fracture energy was increased by 100% (notched sample → healed sample). Therefore, the damage was repaired and some mechanical properties were recovered after healing. Adapted with permission from [82], Copyright 2018 American Chemical Society.

A urethane diacrylate and a linear semicrystalline elastomer were prepared for the 3D printing, which could then be extended by almost 600%. The 3D-printed structures showed outstanding functional properties, for example, a high-strain shape memory with a self-healing ability to heal the microcracks in the printed semi-interpenetrating elastomer network (Figure 6a). Moreover, Figure 6b shows the stress–strain curves of the virgin, notched, and healed samples. Two structural characteristics could contribute to the self-healing capability of the semi-interpenetrating elastomer network: the interdiffusion of the PCL chains and the hydrogen bonding between the urethane groups. As the mechanical property of the elastomer network is low, the recovery of the mechanical properties may be attributed to the interdiffusion of the PCL chains in place of the hydrogen bonding [82].

**Figure 7:**
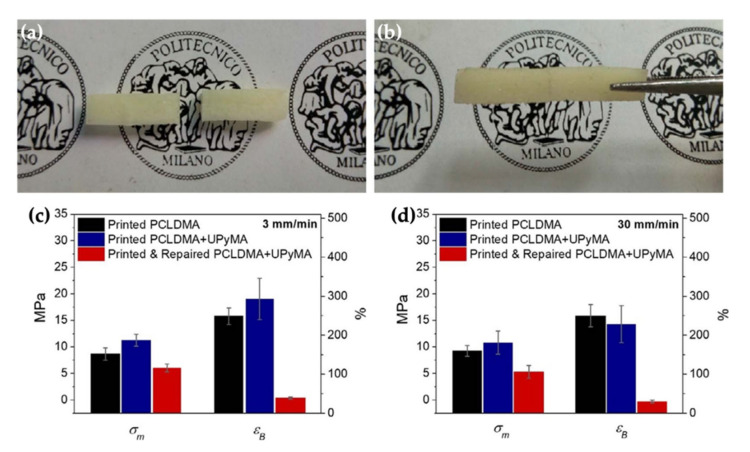
Self-healing results of PCLDMA–UPyMA samples. Images of the cut samples (**a**) and completely repaired samples after the healing process at 80 °C (**b**). Tensile strength (*σ*_m_) and elongation at break (*ε*_B_) of PCLDMA samples (black), PCLDMA–UPyMA samples (blue), and self-healing samples (red), resulted in test speeds of 3 mm/min (**c**) and 30 mm/min (**d**). Adapted from [52], Copyright 2018, with permission from Elsevier.

A 4D-printed shape memory elastomer with self-healing functionalities was thermally triggered and obtained, respectively, by combining polycaprolactone dimethacrylate (PCLDMA) with methacrylates bearing 2–ureido–4[1H]–pyrimidinone motifs (UPyMA). After bulk damage, a great healing performance of two parts of a specimen was obtained after thermal treatment as shown in Figure 7a,b. Moreover, shape memory behaviours were still activated after healing, indicating that this printed SME is suitable for the preparation of biomaterials [52]. A tensile test was utilized to study the mechanical properties of the original and damaged samples. The effect of UPyMA on the mechanical properties of the printed samples was also compared to the printed samples without UPyMA, as in Figure 7c,d.

**Figure 8:**
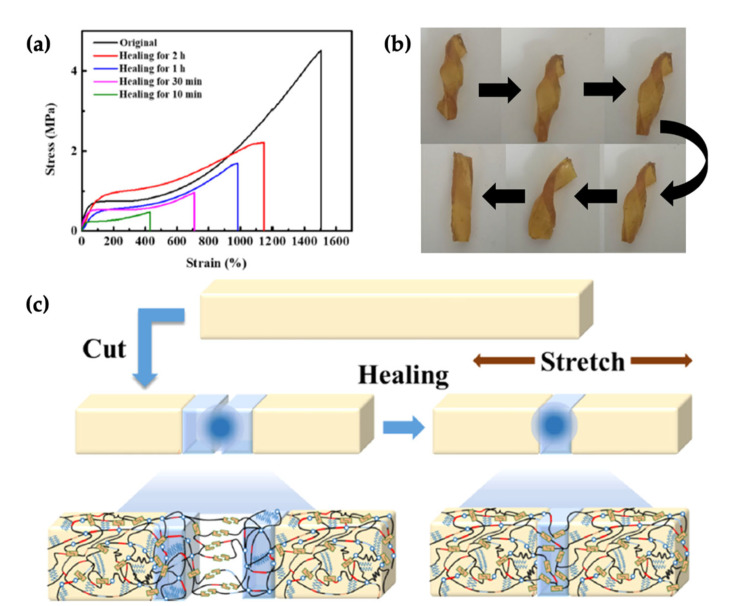
The shape memory with self-healing in biomass bifunctional polyamide elastomers (BbPEs); (**a**) stress–strain curves of BbPEs with various healing times, (**b**) shape memory process of BbPE sample, and (**c**) schematic illustration of the healing process of BbPEs [55]. Adapted with permission from [55]. Copyright 2021 American Chemical Society.

**Figure 9:**
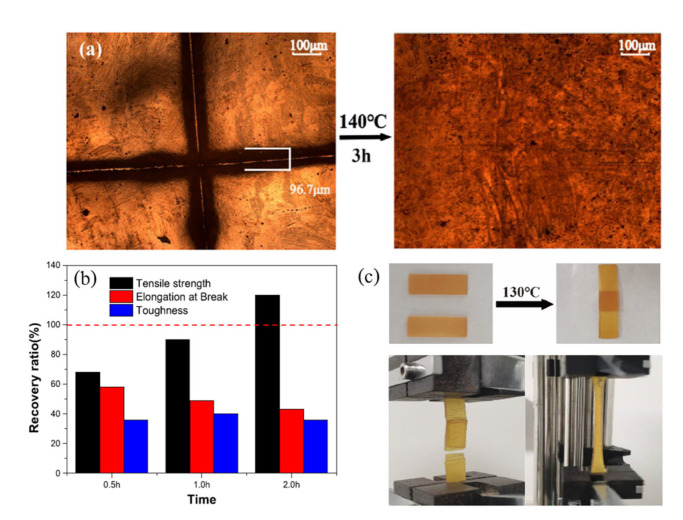
Self-healing of shape memory elastomer from HTPB–PU; (**a**) optical images of self-healing behaviour of HTPB–PUV, (**b**) recovery ratio of mechanical properties of HTPB–PUV fusing at 130 °C for different treating times of 0.5, 1.0, and 2.0 h, and (**c**) optical microscope images of before/after fusing and lap shear testing of HTPB–PUV [86]. Adapted with permission from [86]. Copyright 2022 American Chemical Society.

Chen et al. [55] presented the preparation of biomass bifunctional polyamide elastomers (BbPEs) from dimer acid (DA), trimer acid (TA), and triethylenetetramine to obtain a shape memory behaviour with self-healing characteristics. Figure 8b shows that the sample can be healed within 2 h indicating a healing efficiency of 50%. The results indicated that the longer healing time results in a better healing efficiency due to the TA causing the amide groups between segments to produce chemical crosslinks in the molecules. In the case of the shape memory process, the deformation of a material after 5 min of recovery time returned to about 95% of the original shape. The phenomenon indicated that the internal segments in materials can rapidly respond to temperature changes and accelerate the recovery to their original shape [55]. Furthermore, another example by Li et al. [86] confirmed the effect of temperature on the self-healing process of shape memory elastomers as shown in Figure 9. Hydroxyl-terminated polybutadiene (HTPB)-based polyurethane (PU) networks were prepared as the self-healing samples. They almost recovered from damage after the healing process at 130 °C for 3 h. Therefore, a fusing efficiency of 120% was reached [86].

Furthermore, the indexes for quantifying the self-healing abilities were presented in Buaksuntear et al. (2022) [87]. Generally, the many methods for analysing the self-healing abilities can be summarised as follows: (i) visual aspect of samples, (ii) micrograph analysis using an optical microscope (OM), scanning electron microscope (SEM), or transmission electron microscope (TEM), and (iii) healing efficiency calculations, which are compared to the mechanical properties before and after the self-healing process. For example, the images of the cut samples and completely repaired samples after the healing process are shown in Figure 7a,b [52], and the optical images of the self-healing behaviour of HTPB–PUV are shown in Figure 9a [86], respectively.

## 5. Thermoplastic Shape Memory Elastomers

SMPs also include thermoplastic and thermoset (covalently crosslinked) polymeric materials. Thermoplastic elastomer or TPE is classified as a category of thermoplastic polymer, although it is sometimes referred to as thermoplastic rubber because it is similar in its characteristics and performance to rubber [49,54,88]. The processing of TPE is similar to plastic and it is recyclable. TPEs exhibit high flexibility due to the elastomeric component and they can also be repeatedly extended from their original length at room temperature and then returned to their original shape after stopping the external force.

**Figure 10:**
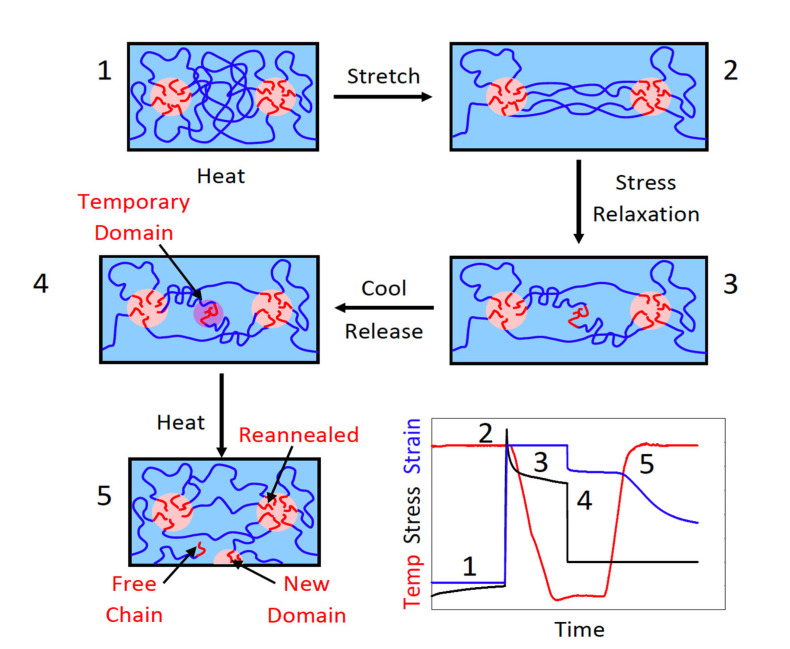
Mechanism of SBCs’ shape memory effect. The stress relaxation started when the sample was cooled down to room temperature (1 → 2). For unloading the sample, the fixed strain increased as the heating time increased (2 → 3). The constant recovery time increased with the increased heating time (3 → 4). This seemed to decrease both the recovery rate and the recovery extent. After heating the sample, it returned to its original state (4 → 5). Reprinted with permission from [49]. Copyright 2019 American Chemical Society.

Styrene block copolymers (SBCs) are a type of thermoplastic elastomers, which include polystyrene–block–polyisoprene–block–polystyrene (SIS), polystyrene–block–polybutadien–block–polystyrene (SBS), and polystyrene–block–poly(ethylene–co–butylene)–block–polystyrene (SEBS) block copolymers. SBCs are commercially available and the required elastomer should be useful in many applications due to its shape memory effect capability such as under changing conditions (e.g., applied strain or temperature). The required SEBS triblock copolymer was developed to induce intrinsic shape memory behaviour. The mechanism of SBCs’ shape memory effect is presented in Figure 10, for which the stress relaxation was investigated at 80 °C for 45 min [49].

Poly(ether–*b*–amide) multiblock copolymers (PEBAs) are a type of thermoplastic elastomer (TPEs) due to their tuned characteristics with soft polyether (SSs) and crystalline aliphatic–amide (HSs). In 2018, Shibasaki and co-workers utilised monodisperse telechelic aromatic N–methylbenzamide-based molecules (MAB_x–x_) with H_2_N–PEG–NH_2_ in high-molecular-weight multiblock poly(*N*–methylbenzamide)–*b*–poly–(ethylene glycol) polymers [poly(MAB_x–x_–*b*–PEG)] using solution polycondensation to make moisture-responsive materials that contain hydrophilic PEG domains. The results revealed three thermal transitions: the glass transition (*T*_g_) and melting (*T*_m_) temperatures of the PEG segment and the *T*_g_ of the MAB_x–x_ segment. The shape memory behaviour was only obtained for the multiblock copolymer films with both crystalline PEG and aggregated monodisperse MAB_x–x_ phases. The co-crystalline structure was necessary for the stability of the temporary shape; the copolymer film returned to its original shape above the *T*_m_ of the PEG phase. After immersion of the film in water for 5 days, it swelled by decreasing the density. The film also exhibited moisture-responsive behaviour after exposing its surface to the humid air [54].

The absorbed water of the film was limited by the MAB phases; when the film was located in an oven, the absorption of the water was fast and the PEG domains formed a crystalline structure that could be returned to its original shape. Therefore, a moisture-responsive material can be developed containing hydrophilic PEG and hydrophobic MAB [54].

**Figure 11:**
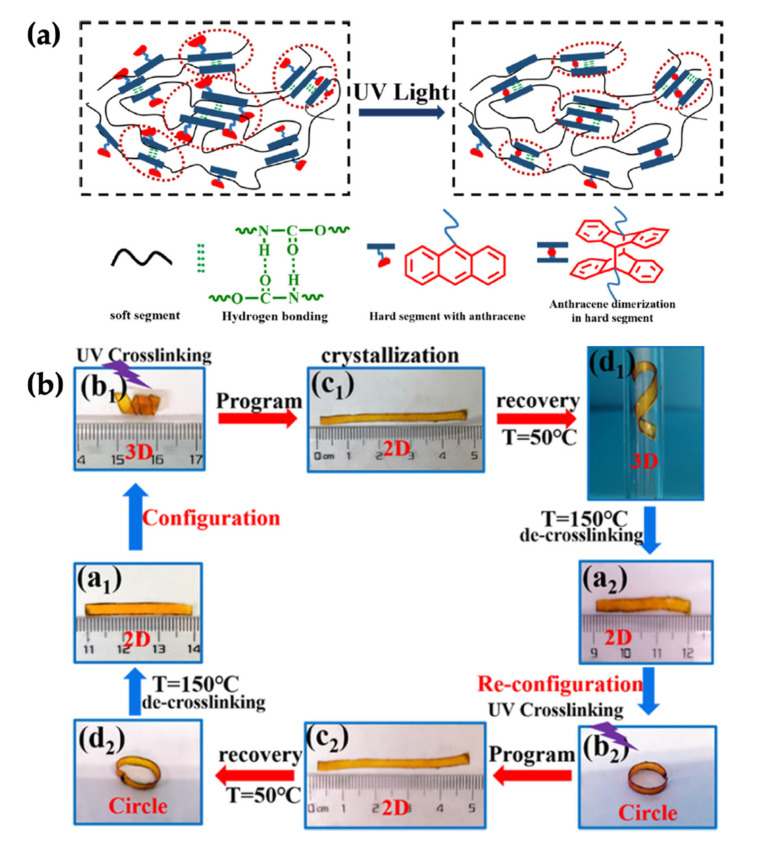
Shape memory thermoplastic polyurethane (TPU) with the photodimerisation of anthracene (AN); (**a**) model of the network structure of AN–TPU networks, and (**b**) shape memory behaviour of AN–TPU samples; (**a1**) is an original shape, (**b1**) is configurated to helix shape using UV irradiation, (**c1**) is fixed to linear shape by crystallization and recovered to helix shape (**d1**) under 50 °C, (**a2**) is de-crosslinking sample under 150 °C, (**b2**) is re-configurated to circle shape using UV irradiation, and (**c2**) is fixed to linear shape by crystallization and recovered to circle shape (**d2**) under 50 °C [47]. Adapted with permission from [47]. Copyright 2021 American Chemical Society.

Normally, thermoplastic polyurethane (TPU) represents a two-phase separated structure that consists of soft and hard segments. Hu et al. (2021) reported that the hard segment via hydrogen bonding played an important role in shape recovery. However, the hydrogen bonding was always weak causing a decrease in the shape recovery effect of the TPU. Therefore, they presented a new type of diol chain extender with anthracene groups incorporated into the hard segment of the TPU to improve the shape recovery behaviour. The results seen in Figure 11 revealed the enhancement of the stability of the hard segments of AN–TPU, indicating the increase in the shape recovery behaviour even at large deformations. Furthermore, the crosslinking density of the elastomer could be controlled by the irradiation time to adjust the shape memory performance [47].

In another example, the shape memory behaviour of ethylene propylene diene monomer (EPDM)/polypropylene (PP) thermoplastic vulcanisates (TPVs) was developed by magnesium acrylate (MgMA). The MgMA reacted both to the PP and EPDM, which increased the compatibilisation of the EPDM/PP blend. The PP phase provided the temporary shape and the EPDM phase induced the recovery to the original shape. The interfacial compatibilisation between the EPDM and PP phases played an important role in the stress passage between the two phases. An appropriate amount of MgMA could be used to increase the crystallinity of the PP phase, which consequently contributed to the shape fixing of the TPV [88].

## 6. Antibacterial and Antifouling Shape Memory Elastomers

Nowadays, the antibacterial and antifouling properties of a material are very interesting as elastomers can be degraded by environmental agents, such as mechanical stress, heating/cooling, chemicals, and hydrolysis under working conditions, causing the polymer to be vulnerable to bacterial contaminations or infections, which are a severe threat to humans. Antibacterial refers to anything that destroys bacteria or suppresses their growth or their ability to reproduce, whereas antifouling systems can be determined as the surface treatment on a substrate to prevent contamination by unwanted organisms [89,90,91].

The hyperbranched polyurethane/sulfur nanoparticles decorated with reduced graphene oxide (HPU/SRGO) were prepared; poly(*ε*–caprolactone) diol (PCL), 1, 4–butanediol (BD), and SRGO dispersion in DMAc were used with the desired amount of xylene. After that, TDI was slowly added to the mixture at room temperature. A tough HPU/SRGO SME represented the shape memory effects under sunlight and exhibited a tremendous enhancement of the mechanical properties. The nanocomposites also showed microbial inhibitory effects against *Escherichia coli, Staphylococcus aureus*, and *Candida albicans*. The HPU only possessed the shape memory property, whereas the SRGO had great energy absorbing ability and good thermal conductivity, which absorbed and then transferred energy to the HPU matrix efficiently. Therefore, the HPU/SRGO showed a great healing performance and the nanocomposites revealed fast self-healing behaviour [90].

**Figure 12:**
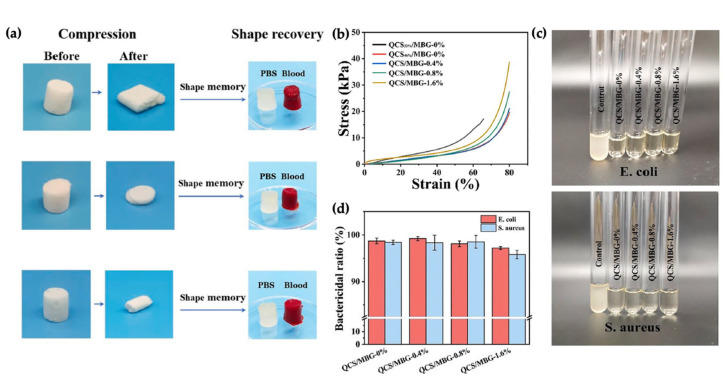
The mechanical properties and antibacterial pursuit of shape memory elastomers; (**a**) the proposed mechanism of shape recovery using blood absorption, (**b**) compressive mechanical properties of QCS/MBG samples, (**c**) *E. coli* and *S. aureus* suspensions incubated with cryogels, and (**d**) bactericidal ratio of cryogels against *E. coli* and *S. aureus* [92]. Adapted from [92], Copyright 2022, with permission from Elsevier.

In another example, Yao et al. (2022) presented shape memory and antibacterial cryogels for clinical application. A shape memory cryogel was obtained via mixing quaternized chitosan (QCS) and mesoporous bioactive glass (MBG) to obtain QCS/MBG cryogels. The results revealed that the mechanical properties depend on the degree of the interconnected macroporous structure. Figure 12a,b show that the cryogels recovered to their original shape after compression, indicating a great compression flexibility and shape memory effect. These relatively great mechanical properties can be contributed to the addition of MBG into the polymer network. The bactericidal ratios of all cryogels were determined by *E. coli* (Gram-negative bacterium) and *S. aureus* (Gram-positive bacterium). Pictures of the bacterial suspensions co-cultured with the cryogels are presented in Figure 12c. Furthermore, Figure 12d shows a high bactericidal ratio for both *E. coli* and *S. aureus* due to the quaternary ammonium groups of QCS that were delivered to the bacterial cell membrane [92].

## 7. Environmental Applications of Shape Memory Elastomers

Petroleum is a fossil fuel that is usually used in transportation and industry; however, it generates global warming. Regarding the UN’s Intergovernmental Panel on Climate Change, petroleum use must cease by the end of the 21st century. Thus, the development of environmentally friendly intelligent materials from renewable sources is urgent.

Poly(lactic acid) (PLA) can be applied to decrease the production of petroleum resources and the emission of CO_2_ during petroleum consumption; however, it also has an appropriate glass transition temperature for SMEs. Polyfuranyl PLAs and polymaleimidyl linkers were reacted to form recyclable shape–memory elastomers. Four-arm hydroxyl-terminated poly(lactic acid) (4HP) and four-arm furanyl-terminated poly(lactic acid) (4FP) were synthesised by the linkers *tris*(2-maleimidoethyl) amine (TMEA) and hexamethylene dimaleimide (HDM). X-ray diffraction (XRD) and dynamic mechanical analysis (DMA) showed that the transition in the shape memory behaviour was induced by the crystallinity of the PLA moiety. The thermal properties of the crosslinked PLA samples were investigated, and the macromonomer 4HP represented the construction and destruction of a crystalline domain. The 4FP-TMEA also established a reversible characteristic in the PLA-based elastomer. These results revealed that recycling the elastomer is possible based on the Diels–Alder reaction observed in this system. These elastomers were also improved in their mechanical properties for disposable products [93].

## 8. Conclusions and Future Research Outlook

In this mini review, we have explored recent developments in processing approaches and the resulting achievable structures, behaviours, and properties of shape memory elastomers (SMEs) suitable for biotechnology applications. SMEs possess unique utilities in comparison to commodity polymers. SMEs are an exciting category of polymeric materials, with the potential to incorporate multi-functionality. Being elastomers, they are soft (low modulus) and can be deformed to large strains and yet recover almost completely. The shape memory aspect enables SMEs to respond to an external stimulus by producing a useful effect such as changing shape and recovering from damage (self-healing). As trigger mechanisms for SMEs can be as creative as the chemical design of the SME, including temperature, pressure/stress, electromagnetic field, moisture/humidity, pH, and solvent, SMEs can be developed and optimised on an application-by-application basis, particularly for biotechnology. In some respects, SMEs can combine the robustness of polymers with the flexibility of hydrogel systems. Indeed, the good hysteresis behaviour and cycle life (temporal repeatability/durability) of SMEs, in comparison to other shape memory polymers (SMPs) is an attractive quality for biotechnology. Moreover, the comparisons of SMEs and shape memory alloys (SMAs) or metals and ceramics were described. The main advantages of SMEs are that they are a soft material, have high flexibility, are lightweight, have good processibility, and have high shape transformations and good recovery compared to the SMAs. The disadvantages of SMEs are the weak recycling process and low thermal conductivity. However, SMEs can be utilised in medical applications [94,95].

Current research in SMEs seems to focus on developing material processes and technologies (without an application in mind at the outset) with limited work then going on to identify applications, develop prototypes, and further enhance and optimise the processes and properties. The latter is an important challenge that needs dedicated attention. In terms of the development of SME material technologies, research has principally explored (i) new and improved processes, (ii) concepts and mechanisms, (iii) material constituents/ingredients, as well as (iv) emerging structures, properties, and multi-functionality. For biotechnology applications of SMEs, we think that specific directions for research would be very welcome.

In terms of processes, with the advent of low-cost 3D printing and its significant potential in biotechnology, exploring fused-deposition moulding processes with SMEs could be an exciting avenue. This could further help the introduction of specific architectures (e.g., hierarchical structures) to enhance the properties and tailor the form of the shape change (e.g., twisting/rotating, straightening/crimping, etc.) as well as produce precision parts.

Following innovations in shape memory elastomers, new concepts and mechanisms could also be explored. For instance, elastomers with dynamic bonds formed through click chemistry or supramolecular chemistry or even the use of interpenetrating networks and co–networks could produce SMEs with a wider range of properties. The nature of these dynamic bonds is inherently amenable to multi-functionalities, such as self–healing, as well as being compatible with the inherent properties of elastomers such as high-failure strains. The use of dynamic bonds and supramolecular interactions is common in nature and could further enhance the dynamic properties, hysteresis, and recovery behaviour. A range of trigger mechanisms has already been explored in the literature. Furthermore, the programming process for the temperature in the static and dynamic methods in tension mode was used to quantify SMEs in order to find the optimal mechanical point and mechanical properties around the glass transition temperature (*T*_g_).

Although more data and solid structure–property relation analyses are needed, the exploration of stimuli more suitable for biotechnology applications, such as pH, solvent, and indirect heat (through ultrasound and infrared), would be useful. In addition, although most research has explored one-way SMEs (being triggered by a single stimulus) that have one temporary shape, SMEs with a memory of two or more temporary shapes that can be triggered at different levels of a single stimulus (one-way SMEs) or even two stimuli (two-way SMEs) are of particular interest for biotechnology applications. For instance, these would enable a more complex set of action patterns for potential use in biomedical devices such as artificial muscles, soft robotics and deployable structures. An example of the latter is in minimally invasive surgery, where an SME device is to be activated in its temporary, compact shape through key-hole surgery and then stimulated to relax to its original, functional shape.

SMEs can be tailored by altering the ingredients and constituents. Exploring SME composites comprising property-enhancing fillers and reinforcements of nano- to macro-scale is another avenue of further growth in the future. Notably, although the use of a filler may enhance certain properties (e.g., modulus, strength), it may be at the expense of other properties (e.g., cycle life/durability) and hence these would need evaluation. Moreover, the addition of a filler creates an interphase and interfaces—the effects and enhancements of these also require attention.

Finally, with regard to research on the emerging properties of SMEs for biomedical applications, their biodegradability and biocompatiblity (e.g., proliferating bioactivity or drug-eluting performance) would be highly advantageous, for instance, to eliminate the need for additional surgery for the removal of a device. The principal challenges would be in designing and managing this multi-functionality, particularly with regard to the choice of stimulus and execution of trigger mechanisms at will or on demand, as well as balancing other physical properties (such as stiffness), and the rate and degree of recovery/reversibility, as well as the cycle time (e.g., fast response would be needed for biomimetic muscles). Imparting durability and managing temporal changes (cycle life) could also be important for biotechnology applications such as in antibacterial and antifouling SMEs. Indeed, properties such as cycle time and cycle life are rarely tested or reported in the literature.

Although the field of SMEs has a lot of growing to do, with several challenges and areas for further exploration, SMEs are in good shape to meet the required specifications for products in the ever-growing and diversifying markets of healthcare and fitness, ranging from personal health monitoring devices and fitness gear and comfort fit products to soft robotics, prosthetics/orthotics, and biomedicine.

## Figures and Tables

**Table 1:** Key differences between polymers and elastomers relevant to shape memory behaviour.

Polymer	Elastomer
Range from highly crosslinked to no crosslinks	Slightly crosslinked
Range from being flexible to rigid	Flexible and elastic
Wide range of glass transition temperatures	Typically low (sub-zero) glass transition temperature

**Table 2:** Example SMEs and their potential applications.

Elastomer	Trigger	Potential Applications	References
Tetrathiafulvalene polymer networks	Thermal, Light	Thermoactuating smart sunshade and sensors	[43]
Polyurethane	Thermal, pH, UV irradiation	Pressure bandages, Bone tissue engineering, Smart textiles, Self-healing materials, Shielding, Heat-shrinkable films, Stent materials, etc.	[44,45,46,47]
Polybutadiene	Thermal	Self-healing materials, Automobiles, Electronics, Medical equipment, Sports materials, and Shoes.	[48]
Polystyrene	Thermal	Sealants, Coatings, Adhesives, and Automotive parts.	[49]
Poly(*ε*–caprolactone)	Thermal, Water, Light	Water-responsive sensors, Actuators, Medical devices, Self-tightening sutures, Smart stents, and Artificial scaffolds.	[50,51,52,53]
Poly(ether–*b*–amide)	Moisture	Biomedical and industrial applications	[54]
Polyamide elastomers	Thermal	Self-healing materials, Intelligent electronic devices, Bionic materials	[55]

**Table 3:** Growth rate and value of market size of SME products.

Products	Growth Rate	Value (USD)
Sports shoes [60]	31%	2320 million
Bedding [61]	17%	115 million
Thermoplastic elastomers [62]	5.8%	5.1 billion

## Data Availability

No new data were generated for this review paper.
